# Patient-Centered Robot-Aided Passive Neurorehabilitation Exercise Based on Safety-Motion Decision-Making Mechanism

**DOI:** 10.1155/2017/4185939

**Published:** 2017-01-16

**Authors:** Lizheng Pan, Aiguo Song, Suolin Duan, Zhuqing Yu

**Affiliations:** ^1^School of Mechanical Engineering, Changzhou University, Changzhou 213164, China; ^2^Remote Measurement and Control Key Lab of Jiangsu Province, School of Instrument Science and Engineering, Southeast University, Nanjing 210096, China

## Abstract

Safety is one of the crucial issues for robot-aided neurorehabilitation exercise. When it comes to the passive rehabilitation training for stroke patients, the existing control strategies are usually just based on position control to carry out the training, and the patient is out of the controller. However, to some extent, the patient should be taken as a “cooperator” of the training activity, and the movement speed and range of the training movement should be dynamically regulated according to the internal or external state of the subject, just as what the therapist does in clinical therapy. This research presents a novel motion control strategy for patient-centered robot-aided passive neurorehabilitation exercise from the point of the safety. The safety-motion decision-making mechanism is developed to online observe and assess the physical state of training impaired-limb and motion performances and regulate the training parameters (motion speed and training rage), ensuring the safety of the supplied rehabilitation exercise. Meanwhile, position-based impedance control is employed to realize the trajectory tracking motion with interactive compliance. Functional experiments and clinical experiments are investigated with a healthy adult and four recruited stroke patients, respectively. The two types of experimental results demonstrate that the suggested control strategy not only serves with safety-motion training but also presents rehabilitation efficacy.

## 1. Introduction

According to the report of World Health Organization (WHO), in recent years, the proportion of the elderly population continues to increase, and many countries of the world are gradually coming into the aged society [1]. Stroke is one of the leading disabling or lethal diseases among the elderly population in the world and usually causes hemorrhagic or ischemic brain damage, which results in some functional deficits, such as motor, sensory, and cognitive limitations [2, 3]. On clinic, majority (more than 69%) of the stroke patients suffer motion disability with upper extremity in some degree [4]. Approximately 610,000 and 2,000,000 people suffer new stroke each year in the United State and China, respectively [5, 6]. The statistical results (in 2013) present that the limb-motion functional disabilities were a key body among all functional deficits; there were 15.64 million persons with the limb disabilities in China, which occupied 59% of the total disabilities. Long-term limitations of function not only impact the quality of daily life but also cause great physical and psychological suffering to patients. Fortunately, clinical medicine has verified that intensive motion training contributes to the recovery of motor neural function.

Recently, all kinds of motion-rehabilitation training robots have been developed to help stroke patients improve the impaired nervous system and motor control, according to the neural plasticity. Meanwhile, many investigations have verified that robot-aided rehabilitation training presents a positive impact in promoting the motion function. Due to the outstanding advantages for robot-aided rehabilitation in high intensity, automated repetition, and recording the data of the training process, the new neurorehabilitation technology with robots has attracted more attention and been rapidly developed. In terms of the upper-limb rehabilitation robots, many researches have done a great contribution in the fields of mechanism design [7], control algorithm [8–10], rehabilitation evaluation, and clinical trial [11]. Motion-rehabilitation training robots are developed in order to help stoke patients or other limb-motion function disorders to relearn motion skills or normal daily functions with robot-aided motion training. It means that the design of rehabilitation system has to follow the rehabilitation movement mechanism. So, safety and effectiveness of rehabilitation are been strongly addressed during the whole process of rehabilitation system design.

Due to the particularity of the service object, safety plays an important role in the rehabilitation system design, which is to be taken for granted. There are various techniques developed to match this issue from the point of hardware or software design. Barkana et al. have developed a quick-release device, same function to the safety mechanism applied in ADLER [12] and GENTLE/s [13], when the physical safety-related event happens, quickly removing the patient's limb out of the rehabilitation robot [14]. This safety mechanism usually works or serves after the event, not online. It cannot supply effective protection to the training subject under the external collision or sudden twitch. In [15], a new reflex mechanism structure was present, which was developed just like the human conditioned response to the external collision. In addition, some other schemes for the hardware design were also utilized to match the operational safety, for instance, making the end-effector work in a small space [16] or driving with pneumatic muscle [17]. The software-based procedure usually applies different detection and analysis techniques to monitor the operating areas and avoid collision with the outside obstacles, such as the estimated danger index [18], mapped virtual reality (MVR) [19], and verbal feedback [14]. In a summary, the existing safety-based designs with hardware or software mainly focus on the external collision, but not the safety motion of the rehabilitation training.

Furthermore, in clinical rehabilitation treatment, different motion-rehabilitation training is usually adopted according to the characteristics of disease and the stage of the recovery. At the early recovery phase, the limb of the stroke patient without any motion ability, passive rehabilitation exercise is usually employed to improve the motion perception and motor nerve system. In terms of the robot-aided passive neurorehabilitation exercise, one position controller is usually designed to serve the training limb tracking the reference trajectories. Due to the “inactive” peculiarity of passive training mode, many investigations pay more attention to developing the position-based tracking control strategies without considering the subject (another part of the rehabilitation system), and the supplied rehabilitation motion exercise is robot-in-charge mode. According to the principles of clinical training, patient-centered service should be well supplied at each stage of the rehabilitation. Patient is a part of the rehabilitation system, which is dynamic. Therefore, to the robot-aided passive training, the motion training should be adaptively regulated in the motion speed and training region for the safety according to the online condition of the patient, but not just tracking the predefined trajectories. However, the existing designed control systems for robot-aided passive rehabilitation exercise pay little attention to the safety from the point of the patient-centered motion training.

Thus, this research presents a novel motion control strategy for patient-centered robot-aided passive neurorehabilitation exercise from the point of the safety. The safety-motion decision-making mechanism is developed to online observe and assess the physical state of training impaired-limb (PSTIL) and motion performances and regulate the training parameters (motion speed and training region), ensuring the safety of the supplied rehabilitation exercise. The patient is taken as a part of the rehabilitation system, and the training movement is carried out with the patient's cooperation to some extent, which is just as what the therapist does.

## 2. Safety-Motion Decision-Making Mechanism

**Figure 1:**
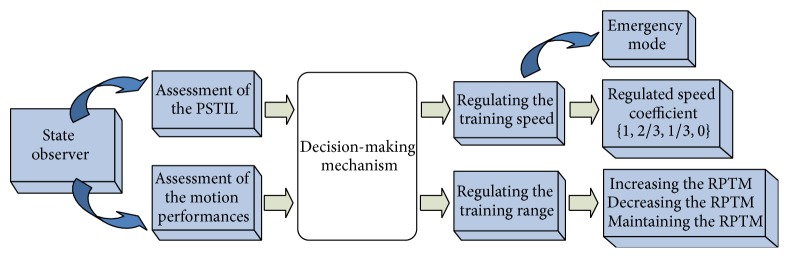
Safety-motion decision-making mechanism.

In clinical therapy, especially at the early stage of the recovery, the safety is strongly addressed when it comes to the passive rehabilitation exercise, because the impaired limb is easy to be hurt. During the traditional hand-to-hand rehabilitation exercise, the therapist usually in real time assesses the PSTIL and motion performances and then draws the impaired limb moving with appropriate speed and training range. The process may be described as observing-and-assessing and decision-making. In this research, the safety-motion decision-making mechanism is developed to play this role, which includes two parts (Figure 1), namely, state observer section and decision-making section. The state observer section in real time assesses the PSTIL and motion performances, and the decision-making section regulates the parameters of motion training to serve with safety rehabilitation.

### 2.1. State Observer

During the passive training, two factors influence the decision for regulating motion parameters. One is the real-time PSTIL, which determines what speed is selected to undergo the current movement. For example, when the patient causes some internal disturbance, such as position-pose changing, and coughing, the training speed should be cut down. The other is the whole motion performances, which reflects the state of the following motion and determines that training range is suitable to the subject. Thus, the state observer is designed to possess the function of assessing the PSTIL and motion performances.

#### 2.1.1. Assessment of the PSTIL

During the robot-aided passive rehabilitation exercise, the impaired limb is relaxed and completely driven by the robot, without applying any active movement. However, the subject is a dynamic part of the cooperation movement, and the PSTIL is usually affected by the internal disturbance of the subject, such as position-pose changing, laughing, talking, and sudden twitch, or by the external disturbance (applying disturbance or collision). In terms of robot-aided passive rehabilitation exercise, it is a typical human-machine interaction activity. It means that, in a sense, the recording data of tracking motion presents the interaction process and further reflects the PSTIL.

During the robot-aided therapy, the motion parameters (position and velocity) are usually recorded. In our previous research [20], the tracking features of position and velocity are extracted and adopted to assess the PSTIL with fuzzy logic reasoning. In order to reflect the dynamic tracking movement effectively, a sliding window is employed to observe the movement information in real time, and the tracking features are extracted with subsection sliding mean square (SMSE). Meanwhile, the variation of the tracking error is also employed with the abstracted feature of subsection SMSE. The final abstracted feature includes the information of the tracking error and the feature abstracted with subsection SMSE, which is described as follows:

(1) χ=αk+λ·femax,emin,αk=∑i=1nx−k−xk−i2n−1,x−k=1n∑i=1nxk−i,femax,emin=emax−e¯×emin−e¯,

where *α*_*k*_ is the abstracted feature of the tracking error corresponding to the *k*th sample data with subsection SMSE, *f*(*e*_max_, *e*_min_) is a function to display the information of the tracking error, *λ* is proportional coefficient, *x*_*k*−*i*_ and ${\overset{-}{x}}_{k}$ are the value of (*k* − *i*)th sample data and the mean of *k*th subsection, and *n* is the length of the subsection window.

According to the abstracted features of position and velocity tracking information, the PSTIL is evaluated with fuzzy logic reasoning. The two features (*χ*_*p*_ and *χ*_*v*_ represent position and velocity tracking, resp.) are managed as the inputs, and the output with fuzzy reasoning reflects the PSTIL.

**Figure 2:**
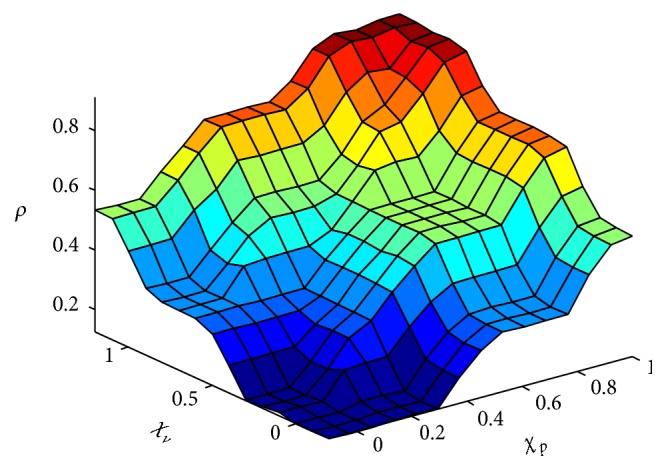
Fuzzy reasoning surface map for assessment of the PSTIL.

In this research, the inputs and output are fuzzified and defuzzfied with five trigonometry membership functions, respectively. According to the designed fuzzy reasoning rules, the overall input-output relationship surface map is shown as in Figure 2.

#### 2.1.2. Assessment of the Motion Performance

Motion performance is the external manifestation of the patient to follow the designed rehabilitation training. It synthetically presents the internal state of the illness, current period status of the patient, and the rationality of the designed task, to a certain extent. Motion performance is associated with the designed exercises. In general, the range of passive training movement (RPTM) is more beyond the patient's capacity more discomfort disturbances caused. In other words, during the passive rehabilitation, motion performance really reflects whether the planed training range is suitable to the patient at that time or not. Therefore, observing the process of following movement and assessing the motion performance play an important role to realize the patient-centered passive rehabilitation exercise. In this research, the motion performances of the current two cycles are observed and assessed online and adopted to guide adjusting the RPTM.

### 2.2. Decision-Making Mechanisms

During the traditional hand-to-hand passive training session, the therapist usually dynamically adjusts the training exercise according to the specific case of the subject at any time. The motion speed and RPTM are two important parameters for rehabilitation training. As mentioned above, the assessments of the PSTIL and motion performance present the impaired-limb state at the execution time and following performance within current period, respectively. Safety is also demonstrated in the rationality and scientificity of the training strategy. When it comes to the robot-aided passive rehabilitation exercise, the movement speed and RPTM should be adaptively adjusted from the viewpoint of safety. Thus, the decision-making mechanisms are formulated to regulate the training motion parameters and realize the patient-centered rehabilitation exercise.

#### 2.2.1. Regulating Motion Speed

During the traditional hand-to-hand motion training, the therapist usually dynamically regulates the stretching speed by evaluating the internal and external condition of the patient, in order to make the training safe. Patient as a part of the rehabilitation training is a dynamic system, which sometimes causes some disturbances for internal or external events. In terms of the robot-aided rehabilitation, when some events (talking, coughing, position-pose changing, sudden twitching, etc.) make the PSTIL changed, it may do some damage to the impaired limb if still stretching the limb with the same speed. Combining the clinical practice, as closely as the therapist, the training speed should be regulated with different degree according to the evaluated PSTIL at that time. When it comes to the emergency events, the robot must do no harm to the patient. In this research, an emergency mode is designed, in which the manipulator moves under full gravity compensation, floating with the arm without any interactive force.

The speed regulation abides by the following rules:

(1) The speed parameter is employed with four grades, namely, {1, 2/3, 1/3, 0}.
(2) In emergency mode, the manipulator moves under full gravity compensation, not stopping.

Online regulating the movement speed according to the evaluated PSTIL effectively enhances the safety of the rehabilitation training.

#### 2.2.2. Regulating Motion Range

In clinical therapy, when the patient undergoes the training motion with suitable RPTM, the therapeutic effect is the best. As mentioned in Section 2.1.2, we can draw a conclusion whether the supplied RPTM is suitable or not by observing and assessing the motion performances of the current two cycles. In this research, in order to serve the patient with more suitable RPTM, the role of the global speed is also considered, when designing the regulating mechanisms. The rules for regulating the RPTM are present as follows.

① During current training cycle, being paused more than 3 times, it means that the undergoing RPTM is beyond the patient's current period capacity and then the RPTM is turned down.
② The global speed planning is also taken into account in the designed regulating mechanisms. When the RPTM is less than [0.35, −0.35] rad in the horizontal exercise or [−5.2, −4.0] rad in the vertical exercise, it means that the patient is in serious condition, and then the global predefined speed should be decreased. The regulating schemes in decreasing RPTM for horizontal and vertical motion exercises are shown in Table 1.
③ In two consecutive motion cycles, there is not any speed regulating or pausing; it means that the RPTM is less than the patient's current period capacity; then the RPTM is increased in the next cycle. Increasing RPTM is in the reverse direction to ②.
④ In other cases, the RPTM is the same as the previous cycle.

## 3. Rehabilitation System and Control System Design

### 3.1. Upper-Limb Rehabilitation System

The Barrett WAM with four degrees of freedom (DOF) is adopted as the main platform to construct the upper-limb rehabilitation system. Barrett WAM has been wildly accepted as experiment platform in the medical field, due to its outstanding dexterity and safety. The WAM is developed with cable-driven technology, which presents good performance in back drivability. Meanwhile, the WAM provides two control panels to do emergency. When pressing the stop button, the end-effector drops not sharply but slowly under gravity, which let the guardian have enough time to deal with the emergency. During the running, the position of each rotary joint is measured and recorded in real time, and the joint can be driven by setting the control torque. The WAM provides an ideal hardware platform to do motion training.

**Figure 3:**
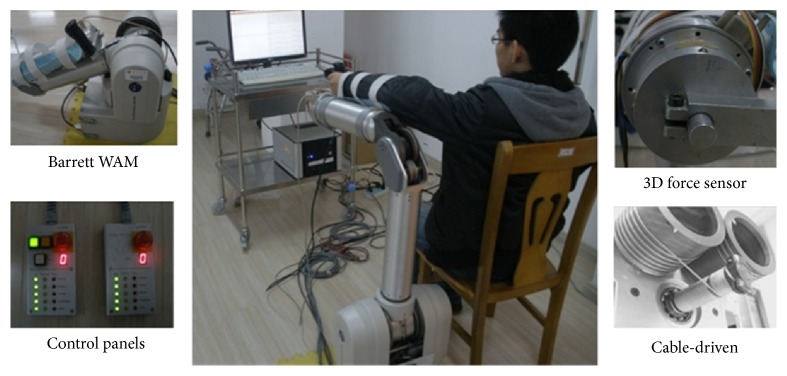
Hardware of the WAM upper-limb rehabilitation system.

According to the requirement of the upper-limb rehabilitation system, a 3D force sensor is developed and installed on the end-effector to detect the interactive force between the impaired-limb and the robot. Meanwhile, in order to support the impaired limb for the seriously ill patients to undergo the passive rehabilitation training, an arm-support device is designed, which could be assembled and disassembled conveniently on the left or right side according to affected side of the subject. The constructed WAM upper-limb rehabilitation system is shown as Figure 3, which mainly consists of the Barrett WAM, external PC, self-developed force sensor, and arm-support device.

The software of the rehabilitation system is developed on the extern PC with Linux system. In order to improve the instantaneity, the real-time module Xenomai is employed. Meanwhile, the tasks of the system are divided into real-time and nonreal-time according to its characteristics, and multithread mechanism is also adopted to manage the tasks.

### 3.2. Control System Design for Patient-Centered Rehabilitation Strategy

Motor learning is widely consented to be a promising method to regain the daily motion abilities for the stroke patient according to the neural plasticity. Passive rehabilitation training is usually introduced to the patient seriously damaged without any motion ability at the early stage of the recovery. The developed robot-aided rehabilitation training should rely on the recovery mechanism [21]. In terms of robot-aided passive rehabilitation, various trajectory tracking control methods are employed to draw the impaired limb following the predefined trajectory. The existing control strategies are usually just based on position control to carry out the training, and the patient is out of the controller. However, to some extent, the patient should be taken as a “cooperator” of the training activity from the viewpoint of patient-centered rehabilitant. Patients with central nerve system (CNS) injured are more vulnerable to injury during the process of movement. Thus, more attention must be paid on the interactive compliance and rationality of the training strategy during the control system design.

Impedance control is firstly developed by Hogan, which describes a relationship between the force and the deviation of the position and velocity [6]. Impedance control is widely adopted to realize the interactive compliance. In this study, the position-based impedance control is selected to execute the compliant following movement.

According to [22, 23] and combining our previous researches [20, 24], the impedance relationship of force and deviation is built with a mass-damper-spring model, described as follows:

(2) ΔF=MdΔX¨+BdΔX˙+KdΔX,ΔX=Xd−X,ΔX˙=X˙d−X˙,ΔX¨=X¨d−X¨,

where Δ*F* is the variance in force, *X*, $\dot{X}$, and $\ddot{X}$ are the actual parameters for position, velocity, and acceleration, *X*_*d*_, ${\dot{X}}_{d}$, and ${\ddot{X}}_{d}$ are the corresponding desired parameters, and *M*_*d*_, *B*_*d*_, and *K*_*d*_ are the desired inertia, damping, and stiffness matrix, respectively.

The position-based impedance control is a method combining the impedance control and position control. The impedance control manages the interactive compliance, and the proportional-integral-derivation (PID) position control guides the upper limb to move along certain paths.

As mentioned in Section 2.2.1, when there is a sudden emergency, the robot works in emergency mode. For this working mode, the manipulator moves under the full gravity compensation, like a feather floating with the arm, not stopping, which effectively protects the training arm under the emergency.

In order to realize the patient-centered rehabilitation, the movement speed and RPTM should be dynamically regulated according to the PSTIL and motion performance, just as what the therapist does in clinical therapy. The safety-motion decision mechanism is developed in Section 2, which plays an important role to serve the subject with safety motion.

**Figure 4:**
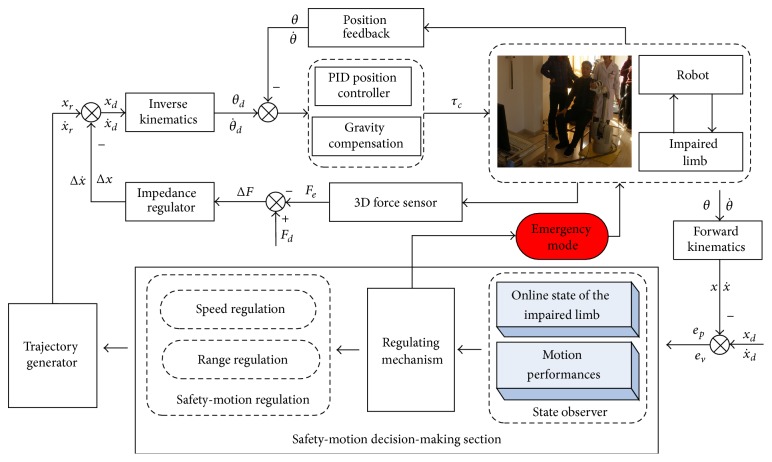
Block diagram of the control system.

The control system with the proposed safety-motion decision mechanism is designed as Figure 4. It mainly includes two sections, namely, the position-based impedance controller and safety-motion decision mechanism. The safety-motion decision mechanism online observes the position-velocity information and assesses the PSTIL at that time and the motion ability at the current period and then regulates the movement speed and RPTM according to the designed regulating mechanisms, supplying decision-making for safety-motion training. The position-based impedance controller is employed to realize the trajectory tracking motion with interactive compliance.

## 4. Experiments and Results

### 4.1. Experiment Scheme

In order to verify the effectiveness and efficacy of the proposed control strategy with the developed safety-motion decision-making mechanisms, the functional experiments and clinical experiments were schemed. A healthy volunteer was guided to carry out the functional experiments. In functional experiments, the subject was asked to deliberately cause some disturbance and make the limb being different PSTIL, to test the regulating function of the developed safety-motion decision-making mechanisms for serving with safety-motion training. Moreover, four stroke patients are recruited to undergo the clinical experiments for investigating the rehabilitation efficacy. The information of the patients is presented in Table 2.

According to the clinical practice, two movement trajectories were predefined, namely, shoulder extension/flexion in horizontal and elbow extension/flexion in vertical. Each type of experiments was carried out with the predefined exercises.

### 4.2. Functional Experiments

**Figure 5:**
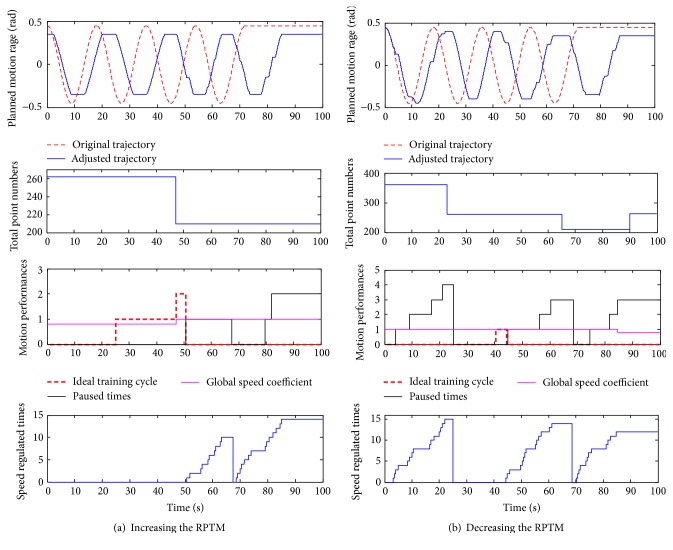
Functional experiments in horizontal exercise.

**Figure 6:**
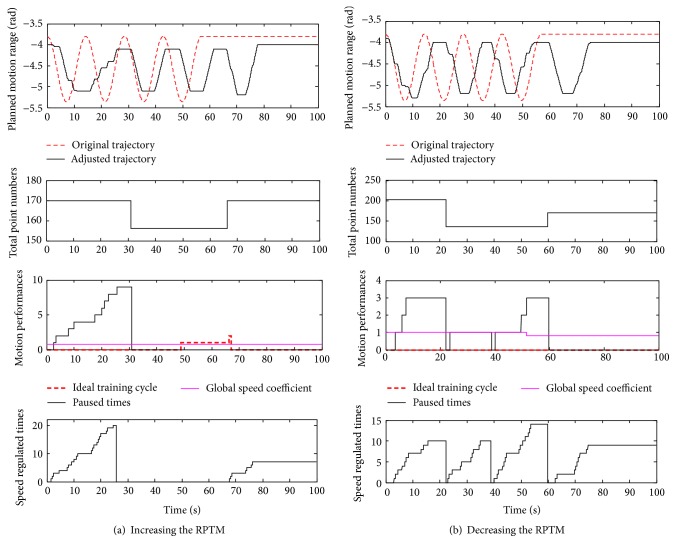
Functional experiments in vertical exercise.

The aim of the function experiments is to test the designed control system with safety motion decision-making mechanism whether it could adaptively regulate the motion parameters (motion speed and RPTM) according to the assessment results online or not. In this type of experiments, the subject is asked to deliberately cause some disturbance just as what may happen in clinical therapy, making the limb being different PSTIL. The disturbance is caused within presenting different regulation function (described in Section 2.2.2). As described in Section 2.2.2, the regulation of the RPTM is based on observing the process of the following motion, which is mainly presented with the speed regulation. Thus, the regulation of the RPTM synthetically reflects the adaptive regulation function in motion speed and RPTM. During the experiments, the corresponding information is recorded to verify the designed functions, such as the number of points in the cycle, the predefined and adjusted trajectories, the speed adjustment or pausing times, number of ideal exercise cycles, and the global coefficient for speed. The functional experimental results of regulating the RPTM in horizontal and vertical exercises are shown in Figures 5 and 6, respectively.

By analyzing the Figures 5 and 6, the designed control system presents a good performance in adaptive regulation the RPTM and global speed coefficient. In Figure 5(a), undergoing two ideal cyclic exercises, it means that the supplied RPTM is less than the subject's capacity, and then the global speed coefficient is increased from 0.8 to 1.0. In Figure 6(b) for vertical exercise, during the first cycle, times of pausing are more than 3, and then the RPTM is regulated down from 203 points to 136 points in the next cycle. Moreover, in Figure 6(b), the predetermined global speed coefficient is regulated from 1.0 to 0.8 in the fourth cycle according to the designed regulating mechanism. In summary, the designed control strategy with safety-motion decision-making mechanism could regulate the motion speed according to the PSTIL and well manage the regulating of the RPTM and the global exercise speed. The safety-motion decision-making mechanism plays an important role in serving with safety-motion training, as what the therapist does in clinical hand-to-hand rehabilitation.

### 4.3. Clinical Experiments

**Figure 7:**
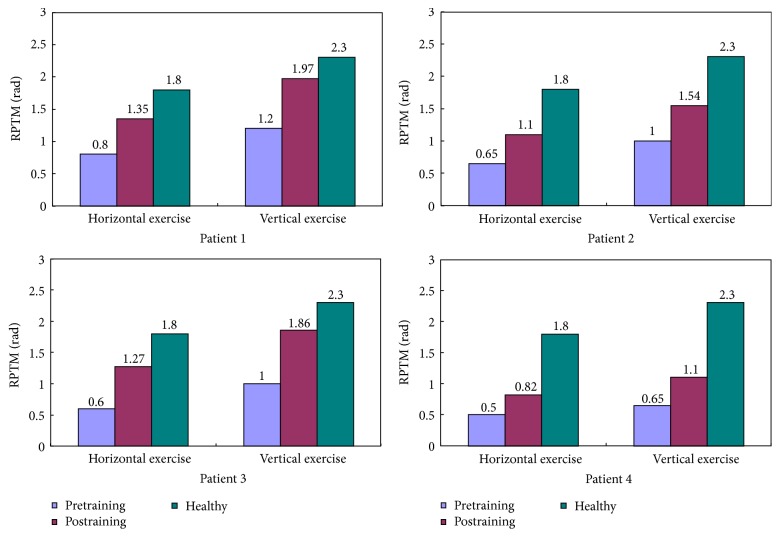
Compared RPTM of the patients for pretraining and postraining.

The aim of clinical experiments is to verify the rehabilitation of the proposed control strategy. Clinical experiments with four recruited stoke patients are carried out with designed patient-centered passive rehabilitation exercise last for one month (22 training days). Each patient undergoes one session in horizontal and vertical exercises, respectively, 30 min/session, one training day. Then comparing RPTM of the patients for pretraining and postraining is shown as in Figure 7. By analyzing Figure 7, each patient regains an increased RPTM, whether in horizontal and vertical exercises or not, and the RPTM of Patient 1 and Patient 3 increase more obviously.

**Figure 8:**
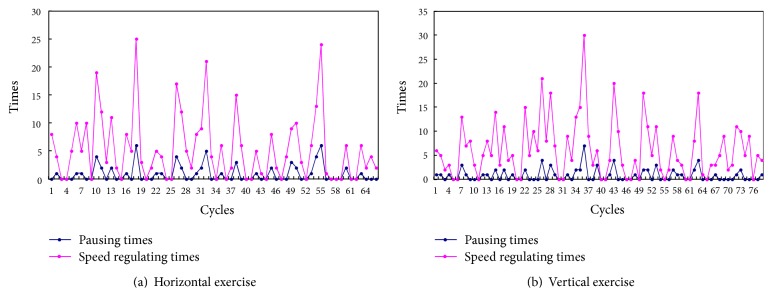
Recorded information of Patient 1 during one session.

**Figure 9:**
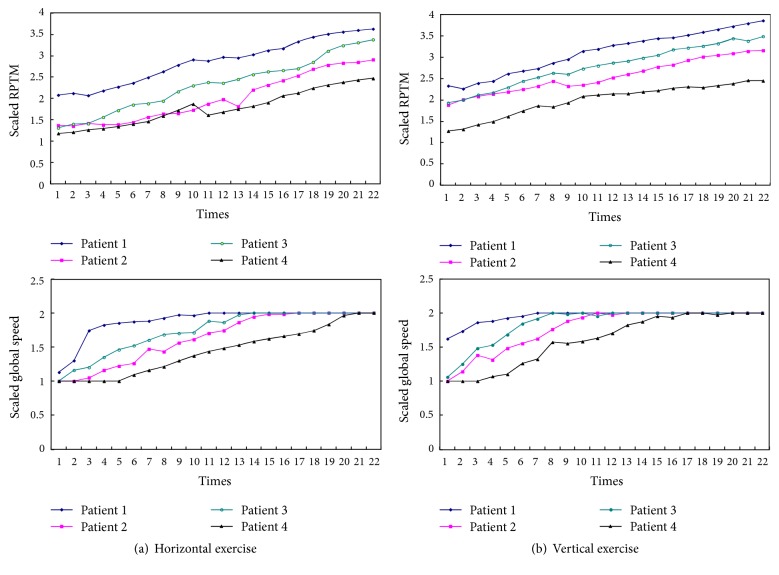
Recorded each mean of the RPTM and global speed (scaled results).

Figure 8 presents the recorded information (times for speed regulating and pausing) of Patient 1 during one session. Because the designed regulation mechanism makes the subject exercise with the maximum suitable RPTM, the times of speed regulating or pausing cannot directly reflect the patient's motion performance. In general, smaller RPTM presents less speed regulating or pausing. The RPTM and global coefficient for speed are determined to the motion performances, so they reflect the motion performances of the impaired limb to some extent. Due to the fact that the RPTM and global coefficient for speed are regulated online, each mean of the parameters is adopted as the comparative indicators. According to the regulation mechanisms in Section 2.2.2, the RPTM is divided into 4 degrees labeled 1~4 and global coefficient for speed into 2 degrees labeled 1 and 2, respectively. Each mean of the RPTM and global coefficient for speed of each training day was described with 22 training days as in Figure 9. By analyzing the Figure 9, a conclusion can be made that the each mean of the RPTM and global coefficient for speed presents rising tendency; in other words, the motion performances of the impaired limb are improved to a certain extent.

## 5. Conclusion and Discussion

In this investigation, a control strategy with safety-motion decision-making mechanism was proposed to realize patient-centered passive neurorehabilitation exercise, serving with safety-and-efficacy robot-aided motion. The safety-motion decision-making mechanism was developed to observe and assess the PSTIL and motion performances in real time and regulate the training parameters according to the internal and external state of the subject, ensuring the safety of the supplied rehabilitation exercise. During the training, the PSTIL was online assessed by fuzzy logic reasoning with the extracted features of the position-velocity tracking information. The movement speed was regulated according to the assessed PSTIL with the designed mechanisms. In terms of safety for the emergency events, an emergency mode was developed, in which the manipulator moved with full gravity compensation. Moreover, the motion performances of the current two cycles were observed and assessed online and adopted to guide adjusting the RPTM according to the designed regulating mechanisms. In order to improve the interactive compliance, the position-based impedance controller was employed to execute the following motion training. Two types of experiments, functional experiments and clinical experiments were schemed and investigated with a healthy adult and four recruited stroke patients, respectively. The experimental results demonstrated that the suggested control strategy not only serves with safety-motion training but also presents rehabilitation efficacy. The developed safety-motion decision mechanism played an important role to serve the subject with safety motion, as closely as what the therapist did. In next work, we will design a series of spatial trajectories, adopt some feedback strategies, and further investigate the rehabilitation efficacy with controlled experiments.

## Figures and Tables

**Table 1:** Scheme for regulating RPTM.

Horizontal exercise	Planning points	360	→	260	→	210	→	262 (0.8v)	→	212 (0.8v)
	RPTM	[0.45, −0.45]		[0.4, −0.4]		[0.35, −0.35]		[0.35, −0.35]		[0.3, −0.3]
Vertical exercise	Planning points	268	→	203	→	136	→	170 (0.8v)	→	156 (0.8v)
	RPTM	[−5.37, −3.8]		[−5.3, −3.9]		[−5.2, −4.0]		[−5.2, −4.0]		[−5.1, −4.1]

Note: 0.8v represents that the global speed is 80% of the predefined speed.

**Table 2:** Information of the stroke patients.

Patient code	Age	Gender	Time since stroke (months)	Impaired limb
1	56	Male	2	Right
2	68	Female	6	Left
3	61	Female	8	Left
4	58	Male	15	Right
